# Circulating microRNA-19a as a Potential Novel Biomarker for Diagnosis of Acute Myocardial Infarction

**DOI:** 10.3390/ijms151120355

**Published:** 2014-11-06

**Authors:** Jianfeng Zhong, Yuan He, Wenjiang Chen, Xiaorong Shui, Can Chen, Wei Lei

**Affiliations:** 1 Department of Cardiovascular Medicine, Affiliated Hospital of Guangdong Medical College, Zhanjiang 524000, China; E-Mails: 15900130806@126.com (J.Z.); yezi62002413@163.com (W.C.); 2 Laboratory of Cardiovascular Diseases, Guangdong Medical College, Zhanjiang 524001, China; E-Mail: yvette.he@hotmail.com; 3 Laboratory of Vascular Surgery, Guangdong Medical College, Zhanjiang 524001, China; E-Mail: shuixiaor@gmail.com

**Keywords:** acute myocardial infarction, biomarkers, diagnosis, miRNA, miR-19a

## Abstract

Acute myocardial infarction (AMI) is a serious cardiovascular disease. Investigating new susceptibility genes for effective methods of early diagnosis of AMI is important. In the current study, peripheral blood miR-19a levels were detected by real-time polymerase chain reaction. Significant differences and logistic correlation analyses were carried out by grouping of disease types and stratification of risk factors. Receiver-operator characteristic curve analysis was used to compare the current common clinical biochemical markers and evaluate the sensitivity and specificity of miR-19a for diagnosing AMI. Circulating miR-19a expression in the AMI group was higher than that in controls. The diagnostic effect of circulating miR-19a levels was superior to current clinical biochemical indices, such as CK, CK-MB, MYO, hs-TnI, and BNP. Our results show that there is a close association of circulating miR-19a levels with susceptibility to AMI. Circulating miR-19a levels could be a candidate diagnostic biomarker for AMI.

## 1. Introduction

Coronary artery disease (CAD), and its most serious complication, acute myocardial infarction (AMI), have become leading causes of death. Reducing the incidence, disability, and mortality of AMI has been a focus of research [1]. Early detection, diagnosis, and early prevention of AMI are required to prevent the progressive development of AMI, and to improve its cure and survival rate in patients [2,3]. Although traditional methods, including an electrocardiogram, coronary angiography, and enzymatic indicators, are important in the diagnosis of AMI, they are insufficient for early detection of AMI [4,5]. Therefore, identifying useful biomarkers for early diagnosis of AMI at biochemical and molecular levels has become a hot topic of current research.

AMI is an inflammatory disease with multifactorial interactions, such as immunization, environmental influences, and genetic factors. The incidence of AMI shows obvious familial aggregation of a large number of genetic susceptibility genes, which play an important role in the development of diseases [6,7,8]. The conditions involved in AMI are complex, and the exact pathogenesis of AMI is still unclear. Therefore, effective diagnosis and treatment of AMI based on susceptible genes remain unclear.

MicroRNA (miRNA) acts as a post-transcriptional regulator at the mRNA level by either degrading the target mRNAs or inhibiting their translation. miRNA has a variety of roles in cardiac and vascular injury and repair, and may reflect the susceptibility to cardiovascular disease [9,10]. Some recent reports have shown that miRNAs are useful markers for the diagnosis of multiple cancers and other diseases [11,12]. Circulating miR-1 was found to be a potential, independent biomarker for diagnosis of AMI based on a clinical investigation of 93 patients and 66 healthy subjects [13]. Larger clinical studies and more effective novel markers are required to develop a technique for diagnosing AMI [13]. An inflammation-related miRNA, miR-19a, plays important roles in vascular homeostasis and inflammatory responses [14,15]. However, little information is available regarding the association of circulating miR-19a and the occurrence of AMI.

Therefore, this study aimed to determine the association of circulating miR-19a levels with the susceptibility of AMI. Our results provide evidence of new biomarkers of clinical diagnosis and could lead to effective candidate prevention strategies for AMI.

## 2. Results and Discussion

### 2.1. Clinical Characteristics of the Study Population

A total of 156 AMI patients and 145 control (Ctl) subjects were studied to determine the association of circulating miR-19a levels with AMI. The baseline clinical characteristics of the study subjects are shown in Table 1.

Significant differences were found in some clinical indices, which are used as risk indicators of AMI at the biochemical level, such as lipoprotein (a) (Lp(a)), blood urine nitrogen (BUN), serum creatinine (Scr), serum uric acid (SUA), blood sugar (Glu), total cholesterol (CHOL), triglycerides (TG), high-density lipoprotein (HDL), low-density lipoprotein (HDL), apolipoprotein A1 (ApoA1), and apolipoprotein B (ApoB) (data not shown). In particular, the biomarkers high-sensitive troponin (hs-TnI), myoglobin (MYO), creatine kinase (CK), CK-MB, and brain natriuretic peptide (BNP) further confirmed the clinical diagnosis of AMI. There were no significant differences in hypertension, hyperlipidemia, and diabetes mellitus between AMI patients and control subjects.

**Table 1:** Clinical and biochemical characteristics of the study population.

Index	AMI	Ctl	*p*
Ages (years)	60.13	58.64	>0.05
Sex (M/F)	125/55	106/63	>0.05
MYO	116.6	59.64	<0.001
hs-TnI	1.04	0.15	<0.001
BNP	917.12	934.57	<0.001
CK	375.64	95.36	<0.001
CK-MB	42.40	17.96	<0.001

### 2.2. Circulating miR-19a Levels in AMI Patients and Their Correlation with Biochemical Indicators

**Figure 1:**
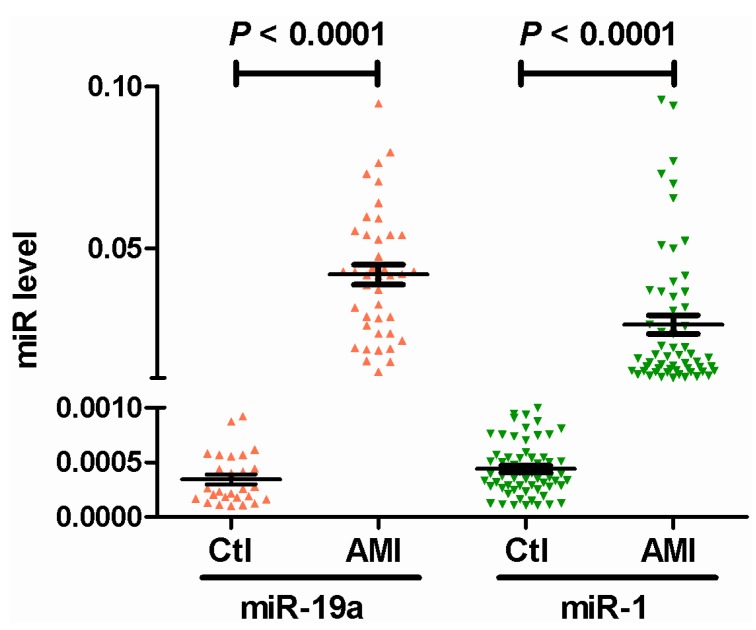
Circulation miR-19a and miR-1 level from acute myocardial infarction (AMI) patients.

Circulating miR-19a levels were approximately 122-fold higher in the AMI group compared with the control group (*p* < 0.0001, Figure 1). In contrast, miR-1 levels were enhanced by only approximately 60-fold in the AMI group compared with the control group, which is lower than the results for miR-19a.

**Figure 2:**
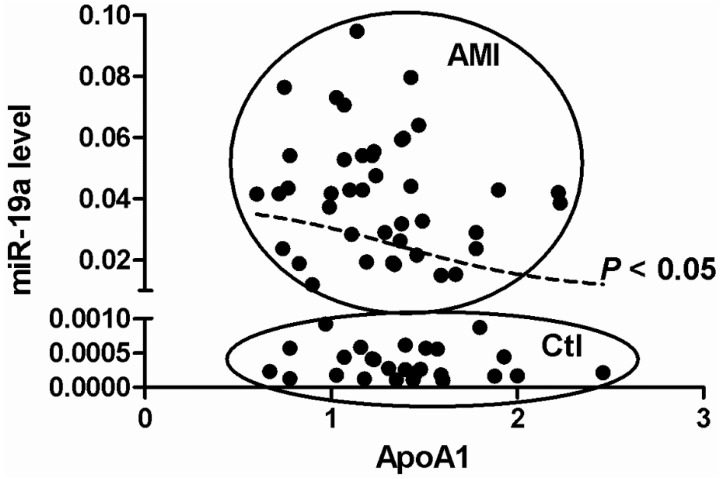
Correlation of miR-19a with the ApoA1.

The odds ratios (ORs) and 95% confidence intervals (CIs) were calculated for the biochemical indicators of AMI, including Lp(a), BUN, Scr, SUA, Glu, CHOL, TG, HDL, HDL, ApoA1, and ApoB (Table 2). We only found a significant correlation between miR-19a and ApoA1 (OR (95% CI): 0.008 (0.000, 0.678), *p* < 0.05), indicating that miR-19a may regulate synthesis and accumulation of HDL by targeting ApoA1 (Figure 2).

**Table 2:** Correlation analysis between miR-19a and the risk factors of AMI.

Group Indicators	Ctl	AMI	*p*	OR (95% CI)
LPa (mg/L)	146.44 ± 21.24	189.36 ± 34.19	0.066	1.007 (1.000, 1.015)
BUN (mmol/L)	5.34 ± 0.24	7.95 ± 2.04	0.605	0.794 (0.332,1.901)
Scr (μmol/L)	68.45 ± 4.93	97.75 ± 4.01	0.313	1.030 (0.972, 1.092)
SUA (μmol/L)	307.74 ± 22.05	355.61 ± 18.98	0.120	1.014 (0.996, 1.031)
Glu (mmol/L)	5.88 ± 0.52	5.94 ± 0.35	0.130	1.370 (0.911, 2.059)
Chol (mmol/L)	4.93 ± 0.15	4.10 ± 0.17	0.087	0.268 (0.059, 1.213)
TG (mmol/L)	1.29 ± 0.16	1.42 ± 0.13	0.272	2.743 (0.453, 16.619)
HDL (μmol/L)	1.62 ± 0.08	1.33 ± 0.09	0.750	0.516 (0.009, 30.205)
LDL (μmol/L)	2.78 ± 0.16	2.39 ± 0.13	0.214	0.349 (0.066, 1.835)
ApoA1 (g/L)	1.39 ± 0.08	1.26 ± 0.06	0.033	0.008 (0.000, 0.678)
ApoB (g/L)	0.86 ± 0.03	1.34 ± 0.49	0.057	7.594 × 10^3^ (0.778, 7.417 × 10^7^)

2.3. miR-19a as a Potential Biomarker of AMI

**Figure 3:**
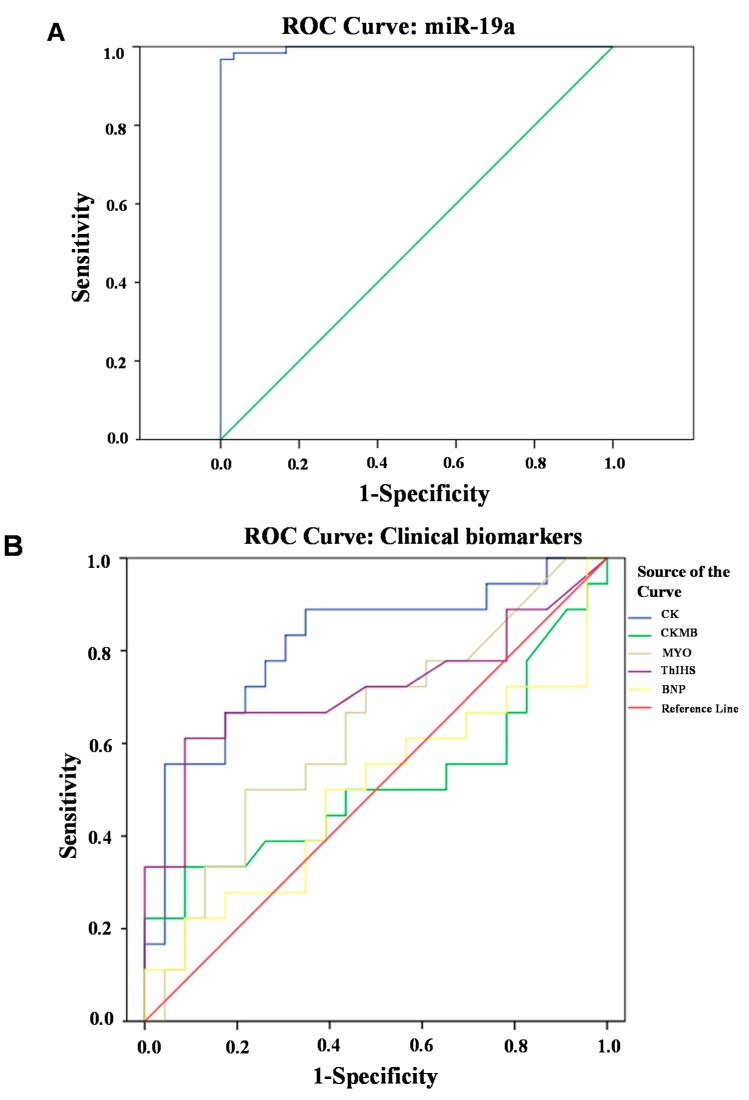
ROC curve analyses of miR-19a and the known biomarkers. (**A**) ROC curve for miR-19a; (**B**) ROC curve for the known biomarkers including CK, CK-MB, MYO, hs-TnI and BNP.

Receiver-operator characteristic (ROC) curve analysis was used to evaluate the predictive power and diagnostic accuracy of plasma miR-19a levels for AMI. After comparing miR-19a levels in the AMI and control groups, the AUC was 0.997 (95% CI = 0.000–1.000, *p* < 0.0001, Figure 3A). When the five biochemical biomarkers CK, CK-MB, MYO, hs-TnI, and BNP were compared, the AUC for CK was 0.812 (95% CI = 0.672–0.951), CK-MB was 0.511 (95% CI = 0.317–0.704), MYO was 0.628 (95% CI = 0.454–0.802), hs-TnI was 0.717 (95% CI = 0.543–0.891), and BNP was 0.493 (95% CI = 0.305–0.681) (Figure 3B). These results showed that miR-19a was a more accurate marker for the presence of AMI than clinical markers that are currently widely used.

Recent evidence has shown that abnormal expression of miRNAs is correlated with progression of various cardiovascular diseases. There is overexpression of miR-19a in many types of cancers, such as inflammatory breast cancer, bladder cancer, and laryngeal squamous cell carcinoma. However, there is little information available regarding miR-19a in AMI studies, especially clinical investigations [16,17,18]. In our study, we found that measuring circulating plasma miR-19a levels was an effective approach for a blood-based molecular assay of human AMI. As an inflammation-related miRNA, miR-19a was significantly higher in peripheral blood of AMI patients compared with non-AMI subjects who only had trace levels of miR-19a in the circulation. Additionally, elevated miR-19a levels were strongly associated with the occurrence of AMI. Consequently, circulating miR-19a levels may be an independent biomarker for diagnosis of AMI, and by analogy, other ischemic myocardial injuries.

To avoid possible sample deviation from selection of clinical subjects, the ages of AMI and controls subjects were limited to 35–75 years, and the sex ratio was also relatively balanced between both groups. Associated complications, including cardiovascular events and inflammatory diseases, were excluded in the present study. Furthermore, logistic regression analysis confirmed that age and sex did not affect miR-19a levels, suggesting that miR-19a as a biomarker is applicable to the broad population. Therefore, miR-19a is a promising and practical biomarker for clinical diagnosis [11,19,20].

Although increasing attention has been paid to the function and applications of miRNA, miRNA has not actually been used in clinical diagnosis yet [21,22]. To strengthen our results, we measured the level of another miRNA, miR-1, which is an important biomarker for AMI. Interestingly, we found that the range in elevation of miR-19a in AMI patients relative to controls was much greater than miR-1. This finding indicated the superiority of blood miR-19a levels as a novel AMI marker.

Some existing biomarkers for AMI, such as CK, CK-MB, MYO, hs-TnI, and BNP, are considered as the biochemical criteria of AMI diagnosis, especially at early admission [23,24]. However, the sensitivity and specificity of these biomarkers remain to be further enhanced, and it is difficult to identify new proteomic markers because of a technical bottleneck [25]. Based on the current study, we propose that miRNAs can be used as molecular biomarkers for diagnosis of AMI. Real-time PCR has a high accuracy and resolution, and has been extensively applied in clinical gene tests of some genetic diseases [26]. Therefore, detection of miRNAs based on real-time PCR can provide a remarkably sensitive and specific approach of early evaluation of AMI. Additionally, miR-19a does not correlate with the known biochemical indicators of AMI, except for ApoA1.This finding suggests that miR-19a is an independent biomarker for susceptibility to AMI. Finally, plasma miR-19a levels in AMI patients were 120-fold higher than control subjects and reached a highly detectable level. Therefore, miR-19a levels in the circulation are closely associated with the occurrence of AMI, and possess a highly predictive and discriminating ability.

## 3. Materials and Methods

### 3.1. Study Population

In this study, 156 patients with AMI and 145 healthy subjects were recruited from the Affiliated Hospital of Guangdong Medical College (Zhanjiang, China) during May to August 2013. AMI was diagnosed angiographically with at least 50% stenosis in at least one of the coronary arteries, together with physiological examinations, including increased hs-TnI, MYO, CK, CK-MB, and BNP levels. A total of 145 healthy subjects who were controls attended routine medical examinations, and they had no clinical manifestation or medical history of heart disorders, family history of coronary heart disease, or abnormal ECG. Those patients who had any acute or chronic infections, inflammatory diseases, severe liver or renal function defects, malignant tumors, heart failure, arrhythmia, cardiomyopathies, or hematological disorders were excluded.

Written consent was obtained from all of the patients in the study. The study protocol was approved by the Ethics Committee of the Affiliated Hospital of Guangdong Medical College.

### 3.2. Biochemical Assays and Blood Collection

All of the patients underwent a standard clinical examination and biochemical assays. Fasting blood samples were prepared to measure indices that are associated with diagnosis of AMI, such as Lp(a), BUN, Scr, SUA, Glu, CHOL, TG, HDL, HDL, ApoA1, and ApoB.

Hypertension was defined as a systolic/diastolic blood pressure (BP) level ≥ 140/90 mm Hg. Hyperlipidemia and diabetes mellitus were diagnosed according to Glu levels ≥ 11.1 mmol/L and CHOL > 5.17 mmol/L, respectively.

In addition, 2 mL of venous blood was collected from each patient in the morning. Blood was then kept in tubes containing sodium citrate without conservants at 4 °C overnight. The supernatant (plasma) was obtained after centrifugation at 1000× *g* for 40 min at 4 °C.

### 3.3. Isolation of RNA and Real-Time PCR

Small RNA was isolated from 2 mL of plasma using the miRcute miRNA Isolation Kit (Tiangen, Beijing, China) according to the manufacturer’s protocol. The quality and concentration of miRNA were detected in a multi-volume spectrophotometer system (Epoch, Biotek, Winooski, VT, USA). cDNA synthesis was performed with the miRcute miRNA First-Strand cDNA Synthesis Kit (Tiangen, Beijing, China). Subsequently, the miRcute miRNA qPCR Detection kit (SYBR Green) was used in real-time PCR for relative quantification of miRNAs with 5S as an internal control. The PCR primers were synthesized by Sangon Biotechnology (Shanghai, China), and the sequences were as follows: (1) miR-19a: AGUUUUGCAUAGUUGCACUACA; and (2) miR-1: UGGAAUGUAAAGAAGUAUGUAU. The amplification reactions were performed using the LightCycler 480^®^ ІІ real-time PCR System (Roche Diagnostics, Penzberg, Germany). Data were collected and analyzed using LightCycler 480 software SW 1.5 (Roche Diagnostics).

### 3.4. Statistical Analyses

SPSS (ver. 18.0, IBM, New York, NY, USA) and GraphPad (ver. 6.0, La Jolla, CA, USA) software were used for the statistical analyses. Continuous variables are described as the mean ± SEM. Comparison among groups was assessed by the chi-square test or ANOVA, followed by *post-hoc* analysis (Bonferroni’s correction). Differences were considered as significant at *p* < 0.05. The associations between target miRNA and occurrence of AMI or its risk factors were estimated using a multivariate logistic regression model. ROC analyses were performed with miR-19a levels plotted against AMI. The area under the ROC curve (AUC) was calculated to evaluate the predictive and discriminating power of circulating miR-19 alevels for occurrence of AMI.

## 4. Conclusions

This study shows an association between miR-19a levels and the occurrence of AMI. The use of miR-19a levels is a novel and useful approach for distinguishing AMI. Additional multicenter, randomized studies with larger samples are required to develop miR-19a as a blood biomarker for diagnosis of AMI, and even for monitoring progression of diseases and treatment responses.
